# Seasonal Assessment of Supercooling Points for Two Introduced and One Native *Laricobius* spp. (Coleoptera: Derodontidae), Predators of Adelgidae

**DOI:** 10.3390/insects10120426

**Published:** 2019-11-26

**Authors:** Ashley A. Toland, Holly A. Wantuch, Donald E. Mullins, Thomas P. Kuhar, Scott M. Salom

**Affiliations:** Virginia Tech, Blacksburg, VA 24061, USA; hwantuch@oda.state.or.us (H.A.W.); mullinsd@vt.edu (D.E.M.); tkuhar@vt.edu (T.P.K.); salom@vt.edu (S.M.S.)

**Keywords:** supercooling point, cold tolerance, *Laricobius osakensis*, *Laricobius nigrinus*, *Laricobius rubidus*, *Adelges tsugae*, hemlock woolly adelgid

## Abstract

The hemlock woolly adelgid, *Adelges tsugae* Annand, is an invasive insect that threatens hemlock species in eastern North America. Several species from the genus *Laricobius* are predators of *A. tsugae* in its native areas of Asia and the western United States. Two *Laricobius* species have been released as biological control agents: *Laricobius nigrinus* Fender, and *Laricobius osakensis* Montgomery and Shiyake. *Laricobius rubidus* LeConte is an adelgid predator native to the Eastern United States, where it can feed and complete development on *A. tsugae* opportunistically. Laboratory assays were conducted to assess the cold hardiness of these three *Laricobius* species, including two distinct populations of *L. osakensis*, by measuring the supercooling points of each species from November 2016 through March 2017. This information may be useful for choosing the best-suited biological control agent for a particular region to control *A. tsugae*. There was a significant difference between the overall mean supercooling point of *L. rubidus* compared to the other *Laricobius* spp. There were also significant differences of supercooling points between *L. rubidus* and both strains of *L. osakensis* in January, and significant differences between *L. rubidus* and all other strains in February. *L. rubidus* appear better adapted to cold extremes in the eastern U.S. than imported *Laricobius* spp.

## 1. Introduction

The hemlock woolly adelgid (HWA), *Adelges tsugae* Annand (Hemiptera: Adelgidae)*,* is an invasive pest in the native range of eastern hemlock, *Tsuga canadensis* (L.) Carriere, and Carolina hemlock, *Tsuga caroliniana* Engelmann, and has caused considerable hemlock mortality across a large portion of eastern North America [1,2,3]. The spread of this pest within the range of eastern and Carolina hemlocks, however, has been limited by cold temperatures [4]. In the winter of 2014, extreme and sustained cold temperatures occurred in the eastern United States, leading to a drastic, albeit temporary, decrease in *A. tsugae* densities [5,6].

The density of the biological control agent HWA, *Laricobius osakensis* Montgomery, and Shiyake (Coleoptera: Derodontidae) decreased concurrently with its host, HWA [7]. This event came two years after the initial releases of this insect, making it difficult to determine if their lack of establishment was due directly to the extreme temperatures or indirectly due to loss of prey. The populations of another biological control agent native to the western United States, *Laricobius nigrinus* Fender were also negatively impacted by the effects of the polar vortex [8,9]. *Laricobius rubidus* is a native specialist predator of the pine bark adelgid (PBA), *Pineus strobi* (Hartig) (Hemiptera: Adelgidae) [10,11,12,13]. *Pineus strobi* is an herbivore specialist of eastern white pine, *Pinus strobus* [11]. As such, both *P. strobi* and *L. rubidus* are distributed throughout the range of *P. strobus* [12,14]. Because *P. strobi* appears to experience negligible winter mortality [13] while *A. tsugae* shows much higher susceptibility to low temperatures [5,6], it is possible that their respective predators may also express varying degrees of cold hardiness.

Most overwintering insects can be described as either (i) chill-susceptible, (ii) freeze-tolerant, or (iii) freeze-intolerant or freeze avoidant. Chill-susceptible insects perish before freezing and are likely found in temperate areas [15,16]. Those that are freeze-tolerant are able to survive internal ice formation through the regulated freezing of their body water, with ice formation potentiated by the action of ice nucleating agents and ice nucleating proteins [17,18,19,20]. Freeze intolerant insects die upon solidification of bodily fluids and require physiological depression of their freezing point. This lowered freezing point is referred to as the “supercooling point” (SCP) and can be measured via detection of the temperature spike that results from the latent heat of fusion within the body [21,22]. To avoid mortality, freeze intolerant insects must maintain body fluid temperatures below their melting points. Several strategies are utilized to accomplish this: removing ice nucleators that may initiate ice formation, synthesizing antifreeze proteins to reduce the nucleation potential of seed crystals, and accumulating extremely high levels of carbohydrate/polyols cryoprotectants (most often glycerol) that lower crystallization temperature [23,24,25,26]. All of these lower the supercooling point of the insect, and as a result, helps to prevent the formation of ice in the hemolymph or gut [27].

Temperature biology is an excellent way to predict species distribution since cold temperatures are often a limiting factor to their success [28]. When determining the supercooling point, lab reared insects should be acclimated to field conditions, since insects physiologically adapt to changing temperatures, impacting their cold tolerance [29,30]. Cold hardiness is not a constant feature in insects, as it may vary throughout the season and year. As a result, supercooling points need to be measured multiple times throughout the year [31]. Special interest was given to how the supercooling point of *L. osakensis* populations, with parental lineage originating from northern and southern Japan, compared to one another in this study. These populations are thought to have different cold tolerances because they are native to different climatic regions of Japan. As a result, the northern population has been released in northern parts of the United States, and the southern population in southern parts of the United States. Mausel et al. [22] determined the supercooling points of the coastal and inland strain of *L. nigrinus*. The coastal strain of *L. nigrinus* was collected from the Puget Trough area (including British Columbia, Victoria, and Seattle, Washington), which has a mild maritime climate [22]. The interior strain of *L. nigrinus* was collected from the Rocky Mountain area (Coeur d’Alene and Moscow, ID), which has a colder and snowier continental climate [22]. Our study was to further understand the cold-tolerance of biological control agents for *A. tsugae*.

Comparing supercooling points of different *Laricobius* spp. and populations in southwestern Virginia is intended to provide an additional indicator as to which biocontrol agent would be best suited for this region of the country, as it has been found that biological control agents can fail to establish if their introduced range is not appropriately matched [22,32]. The goal of this study was to determine whether low recovery numbers were due to beetles being intolerant at low temperatures or other factors (i.e., lack of primary food source, *A. tsugae*) by comparing the supercooling points of the northern and southern populations of *L. osakensis*, *L. nigrinus*, and *L. rubidus* from November 2016–March 2017. *Laricobius osakensis* and *L. nigrinus* have limited ability to feed on other prey species, making it difficult for them to survive when there are extreme fluctuations in *A. tsugae* populations [33,34]. Since *P. strobi* appears to experience negligible winter mortality [13] and *A. tsugae* shows much greater susceptibility to low temperatures [5,6], we hypothesized that their respective specialist predators might also express varying degrees of cold hardiness.

## 2. Methods

### 2.1. Colony Rearing and Acclimating Laricobius Osakensis and Laricobius Nigrinus to Field Conditions

A total of 284 southern *L. osakensis* F1 adults, 200 northern *L. osakensis* F1 adults, and 200 *L. nigrinus* F2 adults from a population of mixed interior and coastal strains were used in this study. The original southern population of *L. osakensis* was collected in the Osaka region of Japan in 2015 and were reared in the insectary at the University of Tennessee in Knoxville, TN (Table 1). The original beetles of the northern population of *L. osakensis* were collected from the northern mountain areas of Japan in 2015 and reared at the insectary at Virginia Tech in Blacksburg, VA (Table 1). *L. nigrinus* is also reared at the Virginia Tech insectary and is a combination of the coastal and interior strains. At the time of this study, the insectary was focused on rearing interior strain *L. nigrinus* (collected from Moscow, ID in Fall of 2014, plant hardiness zone 6a); however, wild populations of *L. nigrinus* larvae came into the lab on HWA-infested branches used for colony food. This resulted in a mixture of the interior reared *L. nigrinus* with the progeny of coastal strain *L. nigrinus* (Puget Trough area of Washington state, plant hardiness zone 8a) that had been released years earlier.

When *A. tsugae* broke diapause in late fall of 2016, lab reared northern and southern populations of *L. osakensis* and *L. nigrinus* were removed from the Virginia Tech Insectary and placed onto *T. canadensis* trees at the Virginia Tech Prices Fork Research Center (Blacksburg, VA). Adult beetles were placed in fine nylon mesh (104 × 94 mesh/square inch) 1 m × 66 cm × 66 cm cages (MegaView Science, Taichung, Taiwan) that were zip tied to hemlock tree branches at breast height for ease of access. Each month from November 2016–February 2017, twenty individuals of each species and population were collected from the cages for supercooling point measurement, between 11 A.M. and 1 P.M. Due to the low density of *A. tsugae* at this location, branches with high *A. tsugae* densities were placed in small cups with floral foam and zip tied to the branches inside the mesh cages. Ten beetles were introduced into each cage and labeled by species. As *A. tsugae* density decreased in the mesh cages, either branches with higher *A. tsugae* density were added or the branches were clipped, and the beetles were collected and put into new mesh cages with fresh food.

These *Laricobius* spp. were acclimated to environmental conditions at this site for two weeks before the first supercooling point measurements were taken in November 2016. Beetles were collected from mesh cages two days prior to laboratory analysis. Missing or dead beetles were recorded. Beetles collected from field cages were kept in plastic containers without food for 48 h at 0 °C before supercooling point (SCP) analysis [22]. Daily average temperature was recorded throughout the acclimation period and experiment.

### 2.2. Field Collection of Laricobius rubidus

*Laricobius rubidus* adults were collected from Dismal Falls, in George Washington and Jefferson National Forest, Giles County, VA, N 37.19263° W 80.89230°, which lies in plant hardiness zone 6b (USDA Plant Hardiness Zone Map 2012). Beetles were collected from *P. strobi*–infested *P. strobus* trees using a beat sheet. Samples were collected in early and mid-November 2016, mid-December 2016, late January 2017, and mid-March 2017. Inclement weather prohibited sampling in February 2017. Beetles were kept in plastic containers without food for 48 h at 0 °C before SCP analysis [22].

### 2.3. Supercooling Point Measurement

Groups of four *Laricobius* adults were placed in specially constructed thermocouple arenas and placed on an electronic cooling plate (Thermoelectronics; Stir-Kool SK-1, Wilmington, DE, USA) used to generate a controlled slow-freezing environment. The cooling plate was linked to a constant temperature water bath (Isotemp; Fischer Scientific, Waltham, MA, USA) (1:1 u/v H_2_O: 95% EtOH) held at −26 °C. Precision fine wire thermocouples (Type T 0.13 × 0.03 mm copper constantan, Omega Engineering, Inc., Norwalk, CT, USA) were covered with a thin layer of zinc oxide thermal grease (Zinc oxide/type B emersion oil) for improved thermoconductivity. The ideal cooling rate was achieved by stacking different materials for the cooling plate calibration. The first layer was a piece of cardboard, followed by the thermocouple arena, foam insulation, a layer of corrugated cardboard, an aluminum weight, and then enclosed in an insulated cover. The apparatus was calibrated to achieve a maximum low temperature of −25 °C. After placing beetles on the thermocouples, cooling was initiated at approximately 25 °C, declining at a rate of about −0.5 °C/min until the supercooling point was reached [22]. Cool-plate settings were adjusted during the analysis in order to maintain a constant cooling rate of −0.5 °C /min. The supercooling point was recorded for each individual, and the means of each species were later compared. The temperature recording equipment was a PC-based system (Windows 7) with an 8-channel high-speed thermocouple interface (Omega OM-DAQ_USB-2401 Multiple Channel USB Data Acquisition Module). This equipment included connections to eight pairs of 0.0762 mm copper/constantan fine wire thermocouples, and the use of OM-DAQ recording software.

## 3. Statistical Analysis

Supercooling point data were transformed using a Box-Cox transformation. Main factors (month and species) and interactions between factors were analyzed to determine the significance of differences (*p* < 0.05). Means were separated using a standard least-squares model and using a Tukey’s post hoc test. Analyses were performed using JMP Pro software by SAS version 13.1 (Cary, NC, USA).

## 4. Results

**Figure 1:**
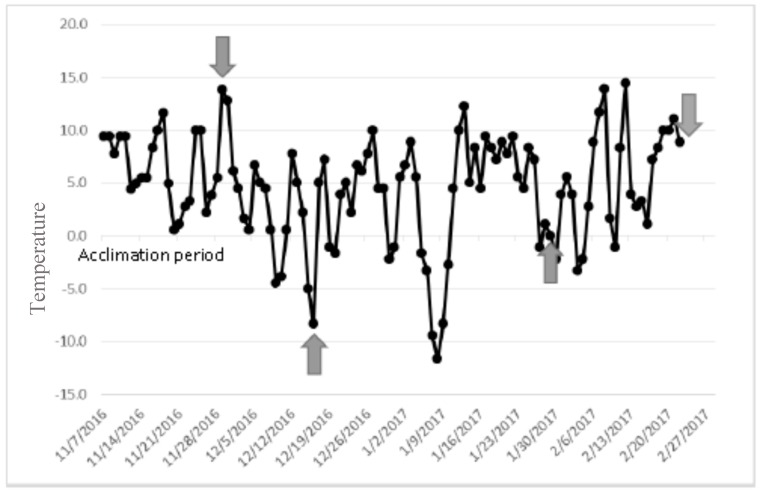
Daily average temperatures in Blacksburg, VA, recorded during the cold hardiness study. The temperatures were plotted as four sampling time intervals: November (7/11/16–28/11/16); December (29/11/16–16/12/16); January (17/12/16–29/1/17); and February (30/1/17–22/2/17), the dates when beetles (N. L.o., S. L.o., L.n.) were removed from the field for the supercooling point (SCP) determination are indicated by arrows.

During acclimation to field conditions, there was a high rate of mortality for all three groups of beetles in the fine mesh cages. The mortality rate of the southern population of *L. osakensis* was 45% (157 adults), the mortality rate of the northern population was 57% (114 adults), and *L. nigrinus* experienced a mortality rate of 52% (104 adults) throughout the course of the experiment. The mean ambient November temperature prior to measurements was 5.5 °C (ranging from 0.6 °C to 13.9 °C), in December was 4.1 °C (ranging from −5.0 °C to 12.8 °C), in January was 3.3 °C (ranging from −11.7 °C to 12.2 °C), and in February was 4.9 °C (ranging from −3.3 °C to 14.4 °C) (Figure 1).

**Figure 2:**
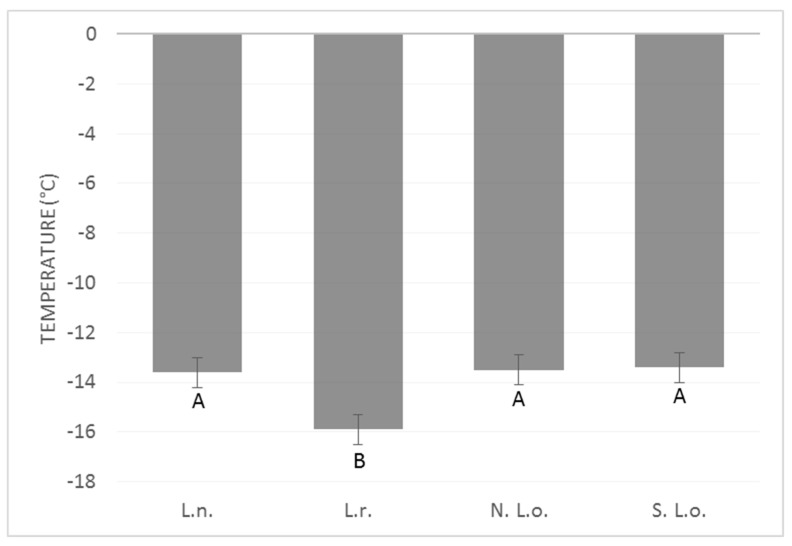
Overall mean supercooling points (± S.E.) of northern *L. osakensis* (N. L.o.), southern *L. osakensis* (S. L.o), *L. nigrinus* (L.n.), and *L. rubidus* (L.r.) measured from November 2016 to February 2017. Error bars indicate standard error.

**Figure 3:**
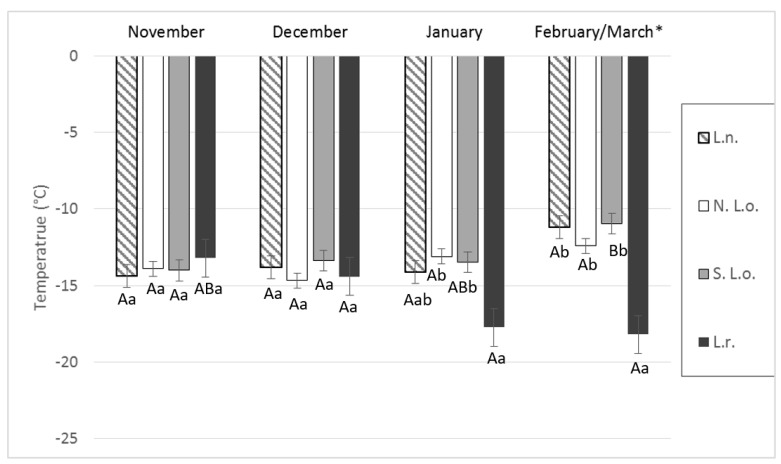
Mean monthly supercooling points from November 2016 to February/March 2017. Species compared include *L. nigrinus* (L.n.), *L. rubidus* (L.r.), northern *L. osakensis* (N. L.o.), and southern *L. osakensis* (S. L.o). Means were separated using a standard least-squares model and a Tukey’s post hoc test. Uppercase letters denote comparison of individual species between months, and lowercase letters denote comparison of species within month. Bars with different letters are significantly different (*p* < 0.05). Error bars indicate the standard error. Total number of insects tested each month were n = 20, except for December (L.r. *n* = 5) and February (L.n. *n* = 13). * *Laricobius rubidus* supercooling points were measured in March; all other groups were measured in February.

The mean supercooling point of the northern population of *L. osakensis* was −13.5 °C, and the SCPs ranged from −5 to −23 °C. The mean supercooling point within the southern population of *L. osakensis* was −13.4 °C, with supercooling points ranging from −5 to −22 °C. The mean supercooling point of *L. nigrinus* was −13.6 °C, with a range of −6 to −21 °C. *L. rubidus* had a mean supercooling point of −15.9 °C (±0.8), with a range of −6 to −27 °C (Figure 2). The mean SCP for *L. rubidus* was significantly lower than both strains of *L. osakensis* in January and significantly lower than all strains/species in February (Figure 3). The southern strain of *L. osakensis* was significantly lower in December than it was in February (Figure 3).

## 5. Discussion

The supercooling points of individual beetles in this study were highly variable, ranging from −5 to −27 °C. It is not uncommon to see high variability in supercooling point studies as there are natural variations of an insect’s cold hardiness from one individual to the next, depending on the time of day and the season [35]. There is also a physiological phenomenon known as rapid cold hardening, which is a short time scale plastic response [36]. In as little as a few minutes, an insect can rapidly adapt to cold temperatures, and this has been shown to impact the supercooling point [37]. This most often occurs when the cooling rate is slower than the recommended rate of about −0.5 °C per minute. Thus, because the rate of cooling was slower than anticipated several times in the study, this could be one explanation for some of the variability found. This 48 hr waiting period was described by Mausel et al. [22] and designed to prevent ingested food from impacting SCP readings. This could potentially be enough time for the insects to physiologically adapt to the conditions of 0 °C when on ice.

The supercooling points of the northern and southern *L. osakensis* population were hypothesized to differ from one another. The average temperatures from November to February in the Nagano region of Japan, where the northern population was collected, ranged between −1 to 7 °C. The average temperatures from November to February in the Osaka region of Japan, where the southern population was collected, ranged between 6 to 14 °C. No significant difference in SCP was observed between cohorts of *L. osakensis*. With this information, future field collections of *L. osakensis* in Japan could be made wherever beetles are most abundant, as opposed to collecting from two separate regions.

The SCP of the *Laricobius* species would be expected to be similar to what Mausel et al. [22] found when they determined the SCPs of the interior and coastal strains of *L. nigrinus* in January of 2008. The average supercooling point from the Mausel et al. [22] study was −16.9 °C (±0.3) for the coastal strain, and between −18.6 (±0.6) and −19.2 °C (±0.7) for the inland strain. The beetles from that study were kept in mesh cages on hemlock trees in Leverett, MA (plant hardiness zone 5a) for four weeks before the supercooling lab work was conducted [22]. These supercooling points are lower than what we found in our study, which was −14.1 °C (±0.9) for *L. nigrinus* in January. Many factors impact the supercooling point of an insect from year-to-year, like beetle health, the locations the beetles were acclimated to, time of year, the fact that Mausel’s study was conducted over three days as opposed to a four-month long assessment, and extreme weather conditions, which could all account for the differences between the results of this study and those of Mausel et al. [22].

There was a high level of beetle mortality in the fine mesh cages. Access to food should not have been an issue since *A. tsugae* were kept at high densities in the mesh cages. The high mortality rate could be a result of the small area of the mesh cages, reducing the ability of the beetles to locate preferred, protected areas when weather conditions were not ideal. The mesh cages may have created microclimates (increased moisture, etc.) with less than ideal conditions for the beetles. Extreme low temperatures in January may have also impacted survivorship, as average daily temperatures went as low as −11.7 °C. Additionally, some of the southern population beetles used for this study emerged from pupation prematurely, which may have contributed to their poor health and high mortality in the first few weeks of the study. Ideally, this study would have continued through March if there had been sufficient numbers of beetles for each species.

The significant differences between *L. rubidus* and the other species could explain why *L. rubidus* populations seemed less impacted by the polar vortex [13]. Pine bark adelgid appears more cold-tolerant than HWA, and based on its comparatively low winter mortality [5,6,13], it follows that their predators could also be more cold hardy. The susceptibility of *L. osakensis* and *L. nigrinus* to cold temperatures may indicate a need to make continuous releases in areas affected by extremely cold weather, such as the polar vortex that occurred during this study.

Our current study was somewhat limited in regard to beetle availability, requiring that lab-reared individuals, albeit after a period of acclimatization, be compared to those collected from wild populations. Additionally, it was not possible to travel to a field site for *L. rubidus* collection in February, and so was necessary to compare individuals collected in March to those of other species collected in February. This may have had some impact on the supercooling points of the field-collected *L. rubidus*, but there was no significant difference between supercooling points in January and March. Thus, it is likely that the delay in field collections had little impact on the overall results. A more direct comparison of supercooling points among *Laricobius* species could be obtained in future studies with more consistent rearing methods and collection times.

Since the first *L. osakensis* releases in 2012 [7], the northern population has been released principally in the northern states impacted by HWA (VA, PA, OH, WV, MD), while the southern population, has been released principally in the southern states HWA (NC, TN, SC). While SCPs are only one measure of adaption to climate, this study suggests that the different species and populations of *L. osakensis* and *L. nigrinus* have similar limitations with cold temperatures. It is not only interesting that the two populations of *L. osakensis* had no significant difference in SCP, despite being collected from very different climates, but also that there was no significant difference between the populations of *L. osakensis* and *L. nigrinus*, as these species were collected from different climates and have evolved in different locations on different species of hemlocks.

## 6. Conclusions

Future work on cold hardiness should focus on the insect’s ability to withstand sustained cold temperatures and the insect’s lower lethal temperature to fully understand the cold hardiness of an insect. Elkinton et al. [38] found that in February 2015, December 2015, and February 2016, the average supercooling point of HWA acclimated at Kentland Farm in Blacksburg, VA ranged from approximately −16 to −18 °C. Despite the year-to-year variation of supercooling points, data suggest that HWA is more cold-tolerant than its predators. This implies that the lack of recoveries for *Laricobius* species after the polar vortex was not only due to the fact that there was a lack of prey to feed on, but also because some of the beetles themselves could not withstand the extreme cold weather. Based on our data, it is likely unnecessary to link release locations of *L. osakensis* to locations they were collected from. *L. rubidus* appear better adapted to cold extremes in the eastern U.S. than the imported *Laricobius* spp., explaining the continued *L. rubidus* recoveries during field seasons during and following the polar vortex. Being native and well-adapted to the region in which *A. tsugae* is invasive, the degree of cold-tolerance exhibited by *L. rubidus* could serve as a standard by which to evaluate the suitability of biological control agents in regard to the survival of winter temperatures.

## Figures and Tables

**Table 1:** Locations where the northern and southern populations of *Laricobius osakensis* adults were collected from in Japan in 2015.

Population	City	Location	Elevation	Number Collected	Plant Hardiness Zone *
Northern	Nagano	N 36.68213, E 138.50032	1720 m	85	7b
Northern	Gunma	N 36.81287, E 139.34099	1550 m	248	8a
Northern	Tochigi	N 36.80325, E 139.42031	1485 m	134	7b
Southern	Nara	N 34.41465, E 135.51145	290 m	2	8b
Southern	Kyoto	N 34.04233, E 135.45733	150 m	14	9b
Southern	Kobe	N 34.44392, E 135.10750	440 m	5	9b
Southern	West of Kyoto	N 34.57570, E 135.36691	375 m	67	9b

* Japan plant hardiness zones based on USDA plant hardiness zone map. www.plantmaps.com/interactive-japan-plant-hardiness-zone-map-celsius.php.

## References

[1] Stadler B., Müller T., Orwig D., Cobb R. (2005). Hemlock woolly adelgid in New England forests: Canopy impacts transforming ecosystem processes and landscapes. Ecosystems.

[2] Nuckolls A.E., Wurzburger N., Ford C.R., Hendrick R.L., Vose J.M., Kloepell B.D. (2009). Hemlock declines rapidly with hemlock woolly adelgid infestation: Impacts on the carbon cycle of southern Appalachian forests. Ecosystems.

[3] Spaulding H.L., Rieske L.K. (2010). The aftermath of an invasion: Structure and composition of Central Appalachian hemlock forests following establishment of the hemlock woolly adelgid, Adelges tsugae. Biol. Invasions.

[4] Evans A.M., Gregoire T.G. (2007). A geographically variable model of hemlock woolly adelgid spread. Biol. Invasions.

[5] McAvoy T.J., Régnière J., St-Amant R., Schneeberger N.F., Salom S.M. (2017). Mortality and recovery of hemlock woolly adelgid (Adelges tsugae) in response to winter temperatures and predictions for the future. Forests.

[6] Tobin P.C., Turcotte R.M., Blackburn L.M., Juracko J.A., Simpson B.T. (2017). The big chill: Quantifying the effect of the 2014 North American cold wave on hemlock woolly adelgid populations in the central Appalachian Mountains. Popul. Ecol..

[7] Mooneyham K., Kok L.T., Salom S.M. (2016). Release and colonization of Laricobius osakensis (Coleoptera: Derodontidae), a predator of the hemlock woolly adelgid, Adelges tsugae. Northeast. Nat..

[8] Heminger A. (2017). Establishment of Laricobius nigrinus (Coleoptera: Derodontidae) in Virginia and Assessment of Its Impact on Hemlock Woolly Adelgid, *Adelges tsugae* (Hemiptera: Adelgidae), throughout the Eastern US. Master’s Thesis.

[9] Sumpter K.L., McAvoy T.J., Brewster C.C., Mayfield A.E., Salom S.M. (2018). Assessing an integrated biological and chemical control strategy for managing hemlock woolly adelgid in southern Appalachian forests. For. Ecol. Manag..

[10] Clark R.C., Brown N.R. (1960). Studies of predators of the balsam woolly aphid, Adelges piceae (Ratz.) (Homoptera: Adelgidae) VII Laricobius rubidus Lec. (Coleoptera: Derodontidae) a predator Pineus strobi (Htg.) (Homoptera: Adelgidae). Can. Entomol..

[11] Doane C.C. (1961). Taxonomy and biology of Pineus strobi (Hartig) and P. coloradensis (Gillette) (Homoptera: Adelgidae). Can. Entomol..

[12] Brown W.J. (1944). Some new and poorly known species of Coleoptera. Can. Entomol..

[13] Wantuch H.W., Kuhar T.P., Salom S.M. (2017). Phenology of the pine bark adelgid, Pineus strobi (Hemiptera: Adelgidae), in white pine forests of southwestern Virginia. Environ. Entomol..

[14] Annand P.N. (1928). A Contribution toward a Monograph of the Adelginae (Phylloxeridae) of North America.

[15] Bale J.S. (1993). Classes of insect cold hardiness. Funct. Ecol..

[16] Sinclair B.J., Alvarado L.E.C., Ferguson L.V. (2015). An invitation to measure insect cold tolerance: Methods, approaches, and workflow. J. Therm. Biol..

[17] Ramløv H., Westh P. (1993). Ice formation in the freeze tolerant alpine weta Hemideina maori Hutton (Orthoptera; Stenopelmatidae). Cryoletters.

[18] Block W., Wharton D.A., Sinclair B.J. (1998). Cold tolerance of a New Zealand alpine cockroach, Celatoblatta quinquemaculata (Dictyoptera, Blattidae). Physiol. Entomol..

[19] Storey K.B., Storey J.M. (2013). Molecular biology of freezing tolerance. Compr. Physiol..

[20] Dunman J.G. (2001). Antifreeze and ice nucleator proteins in terrestrial arthropods. Ann. Rev. Physiol..

[21] Crosthwaite J.C., Sobek S., Lyons D.B., Bernards M.A., Sinclair B.J. (2011). The overwintering physiology of the emerald ash borer, Agrilus planipennis Fairmaire (Coleoptera: Buprestidae). J. Insect Physiol..

[22] Mausel D.L., Van Driesche R.G., Elkinton J.S. (2011). Comparative cold tolerance and climate matching of coastal and inland Laricobius nigrinus (Coleoptera: Derodontidae), a biological control agent of hemlock woolly adelgid. Biol. Control..

[23] Zachariassen K.E. (1985). Physiology of cold tolerance in insects. Physiol. Rev..

[24] Ramlov H., Lee R.E. (2000). Extreme resistance to desiccation in overwintering larvae of the gall fly Eurosta solidaginis (Diptera, Tephritidae). J. Exp. Biol..

[25] Holmstrup M., Hedlund K., Boriss H. (2002). Drought acclimation and lipid composition in Folsomia candida: Implications for cold shock, heat shock and acute desiccation stress. J. Insect Physiol..

[26] Sinclair B.J., Vernon P., Klok C.J., Chown S.L. (2003). Insects at low temperatures: An ecological perspective. Trends Ecol. Evol..

[27] Zachariassen K.E., Kristiansen E. (2000). Ice nucleation and antinucleation in nature. Cryobiology.

[28] Andersen J.L., Manenti T., Sorensen J.G., MacMillan H.A., Loeschcke V., Overgaard J. (2015). How to assess Drosophila cold tolerance: Chill coma temperature and lower lethal temperature are the best predictors of cold distribution limits. Funct. Ecol..

[29] Salt R.W. (1961). Principles of insect cold hardiness. Ann. Rev. Entomol..

[30] Cira T.M., Venette R.C., Aigner J., Kuhar T., Mullins D.E., Gabbert S.E. (2015). Cold tolerance of Halyomorpha halys (Hemiptera: Pentatomidae) across geographic and temporal scales. Environ. Entomol..

[31] Danks H.V. (2005). Key themes in the study of seasonal adaptations in insects I. Patterns of cold hardiness. Appl. Entomol. Zool..

[32] Hopper K.R., Roush R.T., Powell W. (1993). Management of genetics of biological control introductions. Ann. Rev. Entomol..

[33] Zilahi-Balogh G.M.G., Kok L.T., Salom S.M. (2002). Host specificity of Laricobius nigrinus Fender (Coleoptera: Derodontidae), a potential biological control agent of the hemlock woolly adelgid, Adelges tsugae Annand (Homoptera: Adelgidae). Biol. Control..

[34] Vieira L.C., Mcavoy T.J., Chantos J., Lamb A.B., Salom S.M., Kok L.T. (2011). Host range of Laricobius osakensis (Coleoptera: Derodontidae), a new biological control agent of hemlock woolly adelgid (Hemiptera: Adelgidae). Environ. Entomol..

[35] Overgaard J., Sørensen J.G. (2008). Rapid thermal adaptation during field temperature variations in Drosophila melanogaster. Cryobiology.

[36] Teets N.M., Denlinger D.L. (2013). Physiological mechanisms of seasonal and rapid coldhardening in insects. Physiol. Entomol..

[37] Worland M.R., Convey P. (2001). Rapid cold hardening in Antarctic microarthropods. Funct. Ecol..

[38] Elkinton J.S., Lombardo J.A., Roehrig A.D., McAvoy T.J., Mayfield A., Whitmore M. (2016). Induction of cold hardiness in an invasive herbivore: The case of hemlock woolly adelgid (Hemiptera: Adelgidae). Environ. Entomol..

